# Aids to Digestion

**Published:** 1873-01

**Authors:** 


### AIDS TO DIGESTION.

A writer in *Land and Water* reminds the farmer that the more easily food can be digested the more rapidly it will be assimilated to be employed in forming fat and muscle, and in supplying the necessary fuel for keeping up animal heat. In the ruminants, food is, of course, chewed over a second time, and as a rule is more effectually masticated than is the case with horses and pigs. Still, however perfectly mastication may be performed, a good deal of food, especially grain, escapes the grinding action of the teeth, and passes through the stomach and alimentary canal undigested. Proof of this may be observed in grains of oats, which actually germinate after passing through the digestive canal of a horse. An immense saving is effected by bruising, pulping, and crushing all kinds of food given to feeding stock and draught horses, and the only wonder is, when so many clever machines are to be purchased, that the practice is not universally adopted. We are no believers in giving food to animals condensed into a small space; bulk is in all cases essential to healthy action of the digestive organs, but concentrated matters containing highly nutritive properties may be rendered most valuable and efficient, by adopting the easy process of cutting hay and straw into chaff, and mixing it in with the condensed material. Straw especially may be utilized in this way, that under other conditions an animal would not eat. The food is more quickly and easily passed on to be digested the longer time a beast has to sleep and rest, and hence the waste of material consequent upon moving about is economical.

